# Novel Insights into Potential Cannabis-Related Cancerogenesis from Recent Key Whole Epigenome Screen of Cannabis Dependence and Withdrawal: Epidemiological Commentary and Explication of Schrott et al.

**DOI:** 10.3390/genes14010032

**Published:** 2022-12-22

**Authors:** Albert Stuart Reece, Gary Kenneth Hulse

**Affiliations:** 1 Division of Psychiatry, University of Western Australia, Crawley, Perth, QLD 6009, Australia; 2 School of Medical and Health Sciences, Edith Cowan University, Joondalup, WA 6027, Australia

**Keywords:** cannabis, cannabinoids, genotoxicity, epigenotoxicity, carcinogenesis, mutagenesis, oncogenesis, cancer induction

## Abstract

Whilst the cannabis-cancer link has been traditionally described as controversial recent whole nation and whole continent studies have demonstrated that well documented laboratory-based multimodal cannabinoid genotoxicity is indeed reflected in numerous cancer types in larger epidemiological series. A recent longitudinal human sperm epigenome-wide DNA methylation screen in both cannabis dependence and cannabis withdrawal has revealed remarkable insights into the manner in which widespread perturbations of DNA methylation may lead to cancerogenic changes in both the exposed and subsequent generations as a result of both cannabis exposure and withdrawal. These results therefore powerfully strengthen and further robustify the causal nature of the relationship between cannabinoid exposure and cancerous outcomes well beyond the previously published extensive mechanistic literature on cannabinoid genotoxicity. The reported epigenomic results are strongly hypothesis generating and call powerfully for further work to investigate oncogenic mechanisms in many tissues, organs and preclinical models. These epigenomic results provide an extraordinarily close *predictive* account for the epidemiologically observed pattern of cannabis-related malignant disease and indicate that malignant and multigenerational cannabinoid epigenotoxicity is potentially a significant and major public health concern.

## 1. Introduction

The epidemiology of the relationship between cannabis and cancer is often seen as confusing and controversial with both positive [1,2,3,4,5,6,7,8,9,10,11,12] and negative [6,13,14] studies being available. Earlier studies linked cannabis exposure with cancers in adults affecting the brain, head and neck, larynx, lung, prostate, testis, brain, urothelium [1,2,3,4,5,6,7,8,9,10,11] and in several of these studies dose–response relationships were demonstrated [1,3,4,7]. Risk elevation in most studies was between two- and six- fold. Several childhood cancers have also been described following parental gestational exposure to cannabis including rhabdomyosarcoma, neuroblastoma and non-lymphoblastic leukaemia [12,15,16,17,18,19] and such childhood cancers are presumed to be related to inheritable carcinogenic teratogenesis consequent on parental genotoxicity [20,21]. The literature however is controversial with some studies failing to demonstrate a link [6,13]. These studies were reviewed in 2009 by the Californian Environmental Protection Agency who found that six of eleven studies in adults at that time were positive and five of six studies in children were able to confirm a link between parental cannabis exposure and childhood cancer [22].

Provocative new epidemiological studies of community cannabis exposure demonstrate that the cannabis—testicular cancer link [7,23,24,25] has driven the 100% rise in testicular cancer 1975–2018 in USA [26] and is also involved in several common or rapidly growing cancer incidences including breast, liver, thyroid and pancreatic cancer in adults [19] and pediatric acute myeloid leukaemia [16,17,18,19] and fourteen other adult and childhood cancers in USA [27,28,29,30] and Europe [30,31]. Of even greater concern is the recent demonstration that cannabis is driving both acute lymphoid leukaemia the commonest cancer of childhood [32] and also the 50% rise in USA pediatric cancer 1975–2018 [33]. Again these pediatric data raise major issues of transgenerationally heritable teratogenic carcinogenesis [20,21].

More recent epidemiological studies of community cannabis exposure in US have now presented epidemiologically causal linkages between 25 cancers and either cannabis, Δ9-tetrahydrocannabinol (THC) or cannabidiol exposure [27,28,29]. The list of cancers identified was all cancers, acute and chronic lymphoid and myeloid leukaemias, bladder, brain, breast, colorectal, Hodgkins, Kaposi, kidney, liver, melanoma, myeloma, Non-Hodgkins lymphoma, esophagus, ovary, pancreas, prostate, stomach, testis and thyroid [27,28,29].

It is important to note that the genotoxic moiety of cannabinoids has been shown to be the olivetol nucleus on the C-ring [34,35,36] which is shared by many cannabinoids so that cannabinoid genotoxicity/epigenotoxicity is likely to be a class effect shared by numerous cannabinoids.

Moreover, exponential effects for cannabinoid genotoxicity have been well demonstrated on many occasions in the laboratory [37,38,39,40,41,42,43,44] and this finding has been subsequently confirmed epidemiologically [27,28,29].

The group of Schrott and colleagues have recently published an enormously helpful whole epigenome screening study by both whole genome bisulphite sequencing and reduced representation bisulphite sequencing performed in both rats and humans both before and after a 77-day period of documented refraining from cannabis exposure which represents one human sperm cycle [45]. The paper was useful in many ways. It carefully documented functional annotations from Ingenuity Pathway Analysis (IPA) which highlighted cellular development, cell morphology, developmental disorders and nervous system functions during cannabis dependence. Following the period of abstinence cardiovascular system, cell death and survival, nervous system development, organ morphology and organismal death were notable pathways. In both cases the investigators removed cancer-associated annotations apparently because they felt that the IPA was biased towards cancer-related pathways.

The purpose of the present report was to unearth, examine and summarize the cancer-related findings of this noteworthy study and to consider how these remarkable results might fit within the extent published literature both on cellular pathophysiological mechanisms and recent major epidemiological studies.

## 2. Methodology

Data. The source data from the Schrott database [45] relating to DNA methylation changes in semen has been extracted and is provided as a Supplementary File (CaEpi.txt). The genes identified are those which have previously been related to cancer by the research literature.

Analysis. Each mention of the various tumours from the Schrott data appendix was extracted. The *p*-values extracted from the report of Schrott and colleagues was not further processed. Data were grouped and analyzed by the mean, median, minimum and maximum values within each tumour group. These data are presented in Tables and Figures and text. The computational and analytical code in R is also provided as a Supplementary File (translated into MS Word).

The experimental conditions were considered namely overall findings, and findings related to cannabis dependence and cannabis withdrawal considered separately.

Some technical comments are appropriate. Some cancers were not mentioned and were thus unassessable. Gastroesophageal cancers were assigned to both gastric and esophageal classes as their incidence is not dissimilar (about 8 and 5/100,000 according to the Centres for Control (CDC) Surveillance, Epidemiology and End Results (SEER) dataset [46]).

A formal literature review including search terms was not conducted. Rather the focus of this study was on unearthing and explicating the truly remarkable results of Schrott and colleagues and placing them within a conceptual and theoretical position within the mechanistic framework of the published cannabinoid pathophysiological literature.

Ethical Approval. Ethical permission for this study was granted through the University of Western Australia Human Research Ethics Committee on 24 September 2021 with HREC Number 2019/RA/4/20/4724. 

## 3. Results

The 359 pages of Supplementary Material provided along with the paper mentioned cancer 487 times, carcinoma 84 times, neoplasm 28 times, leukemia 32 times and lymphoma 20 times which confirmed that tumourigenesis was indeed a major theme of this dataset. The 176 annotations relating to the 25 tumour types recently identified [27,28,29] may then be extracted and they are shown as Supplementary Table S1 listed by tumour type and by ascending *p*-value.

The data may be summarized by tumour type, the number of annotations, the median and cumulative number of genes referenced and the mean and cumulative *p*-value (Table 1). This table is listed in order of increasing median *p*-values.

The finding that all 20 identifiable cancers which had been linked with cannabis in recent nationwide epidemiological studies and were identifiable in this dataset were positively identified with highly significant differential DNA methylation signals is quite remarkable.

The data may also be divided into findings in cannabis dependence and eleven weeks later in cannabis withdrawal. These comparative data are presented in Table 2. This Table lists the median *p*-value in dependence and the median *p*-value in withdrawal along with their ratio and shows that in most cases the *p*-value in cannabis dependence is much greater, just as the authors note. On the right hand side of this Table appears the numbers of genes annotated in dependence and withdrawal and again notes that the ratio of dependence to withdrawal generally exceeds unity again confirming that the changes of dependence exceed those of withdrawal.

**Figure 1:**
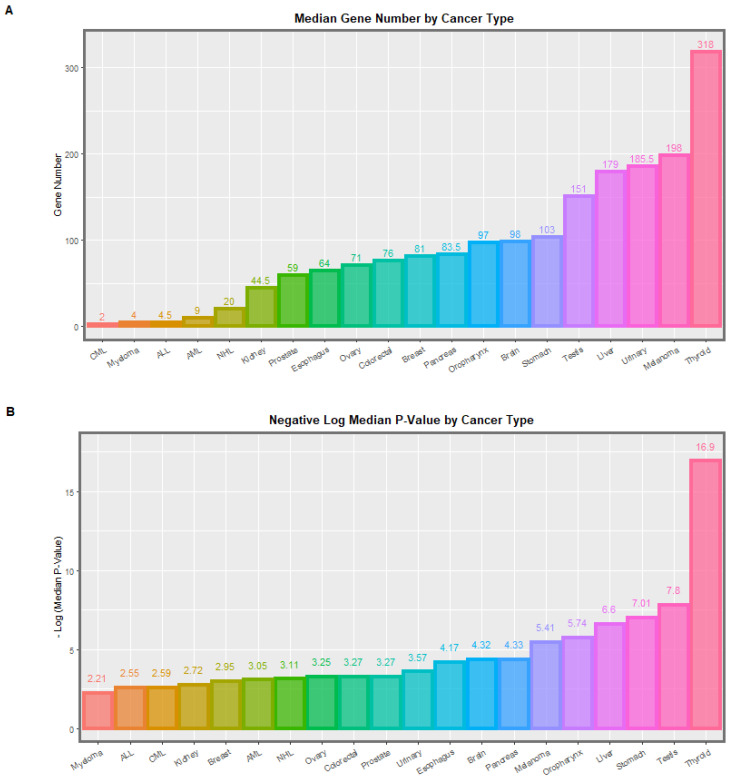
(**A**) Median Numbers of genes annotated and (**B**) median *p*-values for each cancer type for all results overall (from Table 1). Data relate to differential DNA methylation data from sperm for genes previously linked with cancer. The experimental condition considered in this Figure is the overall data (cannabis dependence and cannabis withdrawal considered together). All data from the Schrott data appendix [45].

**Figure 2:**
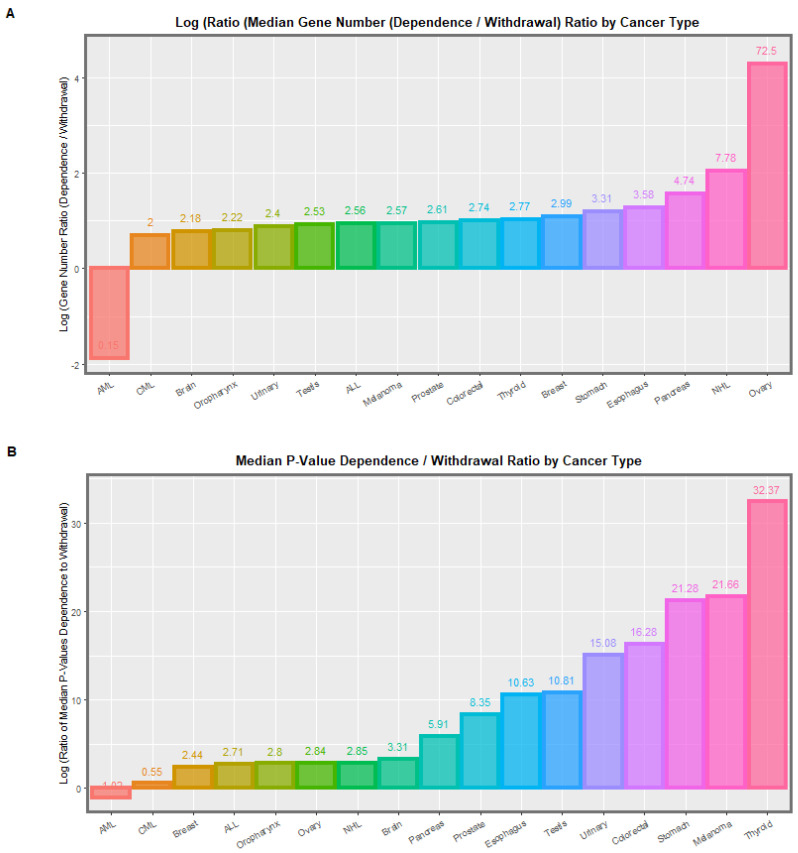
Ratio of (**A**) median numbers of genes annotated and (**B**) median *p*-values in cannabis dependence to withdrawal (from Table 2). Data relate to differential DNA methylation data from sperm for genes previously linked with cancer. The experimental condition considered in this Figure is the ratio of the data in cannabis dependence to that in cannabis withdrawal. All data from the Schrott data appendix [45].

Tabular findings are displayed graphically in Figure 1, Figure 2 and Figure S1. Figure 1 shows the median number of genes implicated and the applicable median *p*-values by cancer type in the whole dataset. Supplementary Figure S1 shows similar metrics in cannabis dependence. Figure 2 shows the ratio of the *p*-values and the gene numbers in cannabis dependence to withdrawal.

As highlighted in Figure 2 the exception to the generality of these observations is the acute myeloid leukaemia [16,17,18,19] where the signal is much stronger in withdrawal than dependence (median 36.0 genes to 5.5 genes; median *p* = 6.26 × 10^−4^ to 0.0017). This is an important finding as some cases of acute myeloid leukaemia occur in early childhood indicating that intergenerational mutagenesis may be at play. This further suggests that in these cases the activation of leukaemogenic gene cassettes by the cannabinoid withdrawal syndrome following birth may actually be activating development of this tumour.

## 4. Discussion

From the perspective of offering a detailed explanation of the diverse pattern of tumourigenesis noted in recent epidemiological studies these results are astounding. They indicate that in both cannabis dependence and cannabis withdrawal DNA methylation changes occur which may in part explain the diverse pattern of tumourigenesis observed both in USA and in Europe.

Cannabinoid genotoxicity however is a aetiopathologically complex involving multiple chromosomal toxicities [39,47,48,49,50,51,52], mitochondrial toxicities [53,54,55,56,57,58,59,60] (which underpin and support the epigenome with substrates and energy amongst other actions) [61], induction of single- and double- stranded DNA breaks [34,36,42,62], oxidation of the bases of DNA [42] and micronucleus induction [62,63,64] and has been reviewed elsewhere [19,27,28,29,45,64,65,66,67,68].

Attributing molecular causal mechanisms may therefore involve parsing out the relative importance of this complex interplay of chromosomal, metabolic, genomic and epigenomic disruptions to properly apportion the importance of the different toxicities which may vary across tissues.

It has been noted that cannabis is often co-administered with other drugs particularly tobacco [69]. Whilst this might perhaps introduce a measure of complexity in epidemiological studies it has been noted by several investigators that tobacco use has been falling in many jurisdictions worldwide whilst cannabis use has been rising [70,71,72] constituting a major trend difference which can be exploited by regression studies and other epidemiological techniques. Moreover, this particular source of confounding has been clarified by the many laboratory studies and the numerous studies in preclinical animal models referenced above.

It is important that cannabinoid genotoxicity has been repeatedly shown to have an exponential dose–response relationship in both laboratory-based metabolic and mutagenic assays [41,42,43,44,73,74,75,76] and in epidemiological field studies [19,27,28,29,66,68,77,78,79].

The findings relating to acute myeloid leukaemia (AML) are intriguing and clearly invite further investigation. Data showed that 6.5 times as many AML-related genes were triggered by cannabis withdrawal compared to cannabis dependence and the median *p*-value fell 2.7-fold. Since some AML cases occur in early childhood (prior to ten years of age) this may imply that the cannabis withdrawal involved in birth transgenerationally triggers early life leukaemogenesis. This hypothesis would need to be tested further experimentally. It could also be tested in other cannabis-related heritable pediatric malignancies including neuroblastoma, rhabdomyosarcoma and acute lymphoid leukaemia [32,80] but is not immediately apparent from the Schrott dataset as these results have been presented.

As study findings are broadly consistent with earlier results from this research group [65] it may be that the present results are broadly generalizable. The clear concordance between the present epigenomic and epidemiological findings provides external validation to these findings and lends further credence to their reliability. It is however important that these results be replicated by other researchers and other laboratories.

It should be emphasized however that whilst the present results are important and intruiging they do not formally demonstrate causality. The present results are strongly hypothesis generating. They do however powerfully call for further research in the laboratory and with animal models to further investigate the intruiging findings reported.

Recently a very powerful single cell RNA sequencing technique which allows the sequential transcriptomic analysis of the same cells across time has been described called “Live-Seq” [81]. Future studies could therefore be envisioned of whole animals and organoids which combine experimentally modelled cannabis dependence and withdrawal with studies of DNA methylation, histone modifications and transcriptomic output with a focus on specific organs and organoids of interest including brain, heart, testis [82] and ovary and the various major cancerogenic tissues. It could be imagined that important mechanistic insights may emerge from such studies including the identification of key genomic vulnerabilities in cannabinoid carcinogenesis and the identification of potential therapeutic targets.

### 4.1. Concise Oncogenic Mechanistic Considerations

Limitations of space necessarily constrain any detailed discussion of the numerous oncogenic mechanisms of cannabis which have been described in detail by previous researchers [19,26,27,28,29,30,32,33,83,84,85,86] however a tightly truncated selection of described effects might be considered as follows.

THC is known to suppress the synthesis of DNA, RNA, proteins and histones [39,40,87,88,89] thereby disrupting the key elements of genomic architecture. Cannabis induces nuclear blebs and chromosomal bridges in sperm, lymphocytes and oocytes changes which are themselves signs of nuclear aging and of major genomic—chromosomal errors [90,91,92]. Cannabis is known to disrupt the replacement of histones by protamines during condensation of the sperm nucleus which necessarily disrupts gene function globally [93,94,95]. Cannabis and several cannabinoids (including cannabidiol) have been shown to oxidize DNA bases which is known to be a potent oncogenic mechanism [42].

Cannabis (including cannabidiol) is well described as causing chromosomal and DNA breaks [62] and tests positively in the dramatic comet assay for DNA breaks (where the broken DNA forms a comet-like tail behind the main mass of DNA moving in a gel under an electrophoretic gradient) [42]. This will lead to breakage-fusion-bridge cycles which is highly oncogenic [96]. This process has been implicated in the rapidly accelerated carcinogenesis occurring in cannabis induced-testicular cancer [26,86]. Cannabis has been shown to induce ring and chain chromosome formation [62] and the formation of microchromsomes and chromosomal circles which form micronuclei which are now known to constitute the central engine of chromothripsis which is a known powerful engine for aggressive cancer formation [64,97,98,99]. Cannabis is known to induce tripolar, quadripolar and higher order mitotic spindles which are associated with disrupted polyvalent cell division [91,92]. Additionally, where a tumour suppressor is silenced or excised or a tumour promoter is aberrantly or constitutively activated by such major megabase scale genomic rearrangements, these changes are a well established pathway to oncogenesis [20,21]. Epigenomic effects can bring a gene enhancer (or superenhancer) into functional contact with a gene promoter and lead to either constitutive activation of a gene promoter or disruption of a tumour suppressor in a manner functionally analogous to that induced by gene rearrangements [20,21].

The vital contribution of RNA exosomes to sperm in the tail of the epididymis (so-called “epididymosomes”) is under endocannabinoid control and is disrupted by exogenous cannabinoids [100]. These exosomes have a critical function in gene expression in the early zygote and impact at least the initial cell divisions. In general global disruption of gene expression is frequently pro-carcinogenic [20,21].

The well described mitochondrial inhibition induced by many cannabinoids (including cannabidiol) [53,54,55,56,59,101,102] will disrupt the supply of energy to the genomic and epigenomic machinery as most reactions involved in genome maintenance are energy consuming. This will directly induce genomic instability which is a well described precursor to malignant change [20,21]. Mitochondrial metabolism also provides many of the intermediate substrates for the epigenomic machinery and signals stress to the nuclear genome via mitonuclear balance [61]. Hence, perturbation of mitochondrial respiration necessarily disrupts normal epigenomic regulation and is pro-carcinogenic. Dozens of other carcinogenic pathways have been described elsewhere [27,28,29,30,103,104].

### 4.2. Strengths and Limitations

There are many strengths of the present study. It is known that the investigative power of the longitudinal design is impressive for epigenomic studies. The epigenomic studies have been performed by a leading laboratory for such work in the world. They are also strongly consistent with major recent epidemiological from USA [27,28,29]. Study limitations relate mainly to the need for these results to be replicated in other laboratories.

## 5. Conclusions

Meanwhile the prior contribution of the Schrott group remains an intriguing and tantalizing data resource to be mined for years to come. The presently reviewed results are strongly hypothesis generating and together suggest much further labotary and preclinical model studies to further investigate the suggested links in an organ-specific manner. Clearly this is a subject which requires extensive future research. Pending such further investigations it is nevertheless prudent from the results described that the strong epigenotoxic findings relating cannabis to malignant disease be carefully considered to understand the strong epidemiological signals which have been reported in relation to cannabis, that cannabinoid genotoxicity be seriously considered as a major matter of public health importance, that the exponential dose response effects of cannabinoids be carefully taken into account, that the food chain be protected, and that appropriate attention be give to the substantial genotoxic and epigenotoxic effects of numerous cannabinoids for the present and subsequent generations. Clearly data imply the need to carefully protect populations from exposure to genotoxic and epigenotoxic cannabinoid compounds of various types. Meanwhile much further research is indicated on these intriguing and important results.

## Figures and Tables

**Table 1:** Overall Significance Levels and Gene Numbers Grouped by Cancer Type.

No.	Cancer	Number Annotations	Median *p*-Value	Median Gene Number	Cumulative Gene Number	Cumulative *p*-Value	Mean *p*-Value
1	Thyroid	3	1.26 × 10^−17^	318	752	0.1622	2.16 × 10^−4^
2	Testis	3	1.60 × 10^−8^	151	364	0.0405	1.11 × 10^−4^
3	Stomach	5	9.77 × 10^−8^	103	545	0.2039	3.74 × 10^−4^
4	Liver	5	2.52 × 10^−7^	179	890	0.0020	2.24 × 10^−6^
5	Oropharynx	3	1.82 × 10^−6^	97	239	8.94E-04	3.74 × 10^−6^
6	Melanoma	4	3.86 × 10^−6^	198	804	0.0080	9.94 × 10^−6^
7	Pancreas	12	4.65 × 10^−5^	83.5	881	0.7067	8.02 × 10^−4^
8	Brain	28	4.74 × 10^−5^	98	3726	0.2777	7.45 × 10^−5^
9	Esophagus	7	6.80 × 10^−5^	64	582	0.2755	4.73 × 10^−4^
10	Urinary	10	2.69 × 10^−4^	185.5	1870	0.4457	2.38 × 10^−4^
11	Prostate	7	5.33 × 10^−4^	59	557	0.3877	6.96 × 10^−4^
12	Colorectal	18	5.34 × 10^−4^	76	2186	0.8214	3.76 × 10^−4^
13	Ovary	7	5.62 × 10^−4^	71	530	0.2669	5.04 × 10^−4^
14	NHL	15	7.77 × 10^−4^	20	365	0.3047	8.35 × 10^−4^
15	AML	3	8.96 × 10^−4^	9	47	0.0475	0.0010
16	Breast	10	0.0011	81	851	0.7765	9.12 × 10^−4^
17	Kidney	2	0.0019	44.5	89	0.1773	0.0020
18	CML	8	0.0026	2	22	0.0585	0.0027
19	ALL	14	0.0028	4.5	141	0.2507	0.0018
20	Myeloma	3	0.0062	4	10	0.0398	0.0040

Table key: The experimental condition considered in this Table is the overall results (cannabis dependence and withdrawal combined). Cancers were grouped by cancer type. The table lists the various classes of gene numbers and *p*-values as described in the column headings. All data is taken from results reported from the epigenomic data appendix in the study of Schrott and colleagues as referenced [45].

**Table 2:** Significance Levels and Gene Numbers Grouped by Cancer Type and Cannabis Dependence or Withdrawal Status.

Cancer	*p*-Values			Gene Numbers		
	Median *p*-Value Dependence	Median *p*-Value Withdrawal	*p*-Value Ratio	Median Gene Number Dependence	Median Gene Number Withdrawal	Gene No. Ratio Dependence/Withdrawal
Thyroid	1.24 × 10^−17^	0.0014	1.14 × 10^14^	318.5	115.0	2.77
Melanoma	1.36 × 10^−14^	3.49 × 10^−5^	2.56 × 10^9^	289.5	112.5	2.57
Stomach	1.53 × 10^−12^	0.0027	1.74 × 10^9^	169.0	51.0	3.31
Colorectal	7.38 × 10^−11^	8.67 × 10^−4^	1.17 × 10^7^	197.0	72.0	2.74
Urinary	1.12 × 10^−10^	3.94 × 10^−4^	3.53 × 10^6^	268.0	111.5	2.40
Testis	1.37 × 10^−8^	6.75 × 10^−4^	4.93 × 10^4^	152.0	60.0	2.53
Esophagus	4.89 × 10^−8^	0.0020	4.14 × 10^4^	136.0	38.0	3.58
Liver	2.52 × 10^−7^	−	-	179.0	-	-
Prostate	8.39 × 10^−7^	0.0036	4.24 × 10^3^	128.0	49.0	2.61
Oropharynx	9.73 × 10^−7^	1.60 × 10^−5^	16.45	97.5	44.0	2.22
Brain	5.82 × 10^−6^	1.60 × 10^−4^	27.38	179.0	82.0	2.18
Pancreas	1.65 × 10^−5^	0.0061	368.39	92.5	19.5	4.74
NHL	2.08 × 10^−4^	0.0036	17.33	35.0	4.5	7.78
ALL	2.23 × 10^−4^	0.0034	15.06	11.5	4.5	2.56
Breast	3.45 × 10^−4^	0.0040	11.51	127.0	42.5	2.99
Ovary	4.10 × 10^−4^	0.0070	17.12	72.5	1.0	72.50
AML	0.0017	6.26 × 10^−4^	0.36	5.5	36.0	0.15
CML	0.0018	0.0031	1.74	4.0	2.0	2.00
Kidney	0.0019	-	-	44.5	-	-
Myeloma	-	0.0062	-	-	4.0	-

Table key: This Table considers the experimental conditions of cannabis dependence and cannabis withdrawal separately. Cancers were grouped by cancer type. The table lists the various classes of gene numbers and *p*-values and their ratios as described in the column headings. All data is taken from results reported from the epigenomic data appendix in the study of Schrott and colleagues as referenced [45].

## Data Availability

All data generated or analysed during this study along with the relevant computational code in R are included in this published article and its supplementary information files.

## Supplementary Materials

The following supporting information can be downloaded at: https://www.mdpi.com/article/10.3390/genes14010032/s1. Table S1: Input data extracted from source data in the Schrott et al. technical appendix (reference [43]). Input Data for Analysis (as text file.txt named “CaEpi.txt”) which is easily convertible into a.csv file in MS Excel. R code for analysis (supplied as an MS Word file but easily copied into an R Script). Named “R_Code for Cancer Epigenomics Paper”. Figure S1: (A) Median Numbers of genes annotated and (B) median *p*-values for each cancer type for cannabis dependence (from Table 2).
