# Supplementary material for: Novel Insights into Potential Cannabis-Related Cancerogenesis from Recent Key Whole Epigenome Screen of Cannabis Dependence and Withdrawal: Epidemiological Commentary and Explication of Schrott et al
Source: Genes (Basel). 2022 Dec 22;14(1):32. doi: 10.3390/genes14010032 (PMC9858221; doi:10.3390/genes14010032)
Supplement: Supplementary file 1 [file genes-14-00032-s001.zip › genes-2067271-Supplementary.pdf]

Concise Description of Supplementary Materials

CaEpi.txt - Source file for R for computations

R_Code for Cancer Epigenomics Paper.docx - R Code for computations for graphs and Tables.

Supplementary Table 1_Canc_Epigenom.docx - Supplementary Table 1.: List of epigenomic hits by cancer type from Schrott et.al. database (Reference [43]).

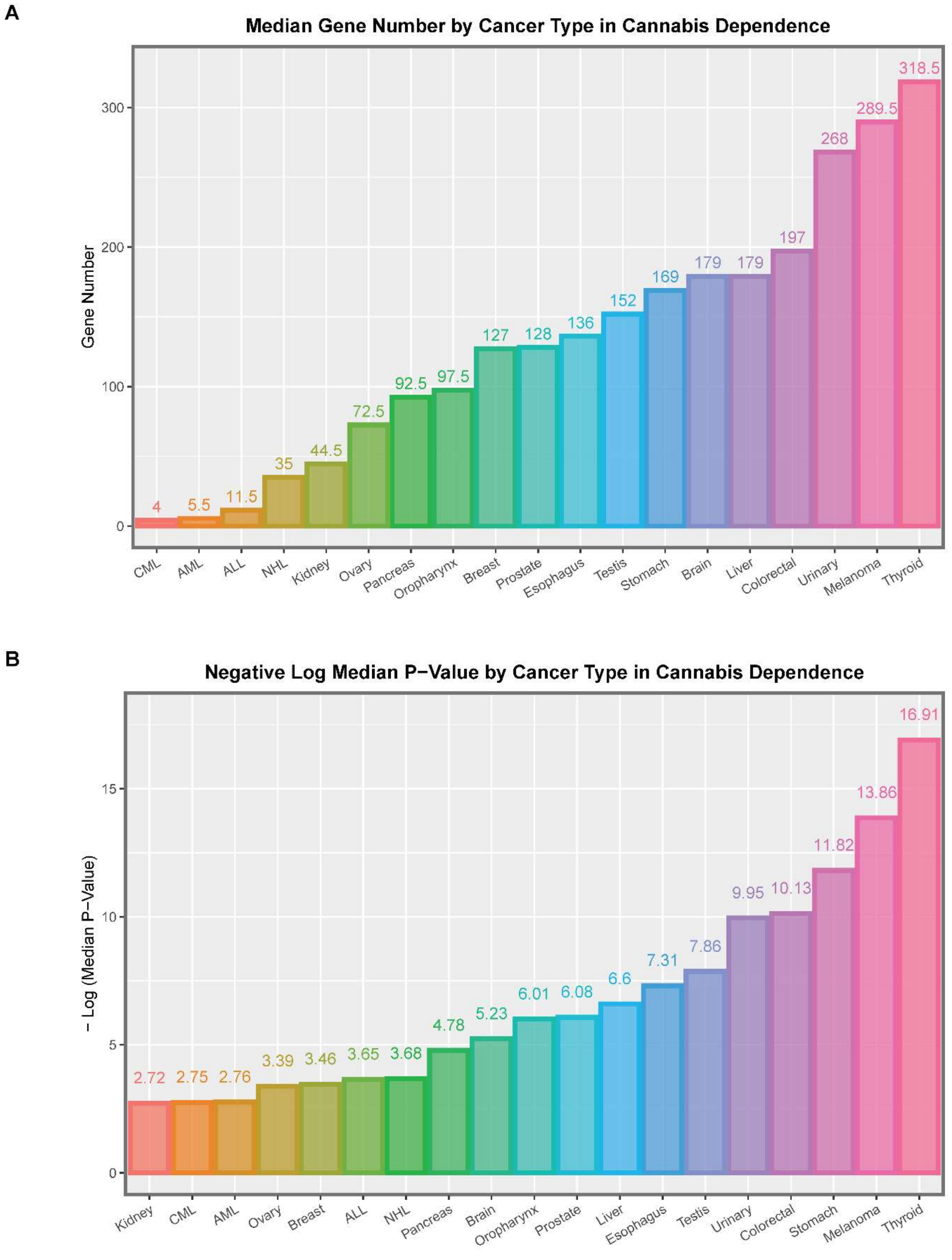

Figure S1: (**A**) Median Numbers of genes annotated and (**B**) median *p*-values for each cancer type for cannabis dependence (from Table 2).

Supplementary Table S1.: Schrott et.al. Cancer Annotation Data Re-Formatted

Cancer	Cancer_Short_Name	Cannabis Dependence / Withdrawal	Page	P-Value	Number of Genes
Thyroid	Thyroid	Dependence	241	1.21E-17	318
Thyroid	Thyroid	Dependence	242	1.26E-17	319
Thyroid	Thyroid	Withdrawal	345	0.001410	115
T-Lymphoblastic Leukaemia / Lymphoma	ALL	Dependence	300	4.08E-05	20
Philadelphia Chromosome Positive Chronic Myeloid Leukaemia	CML	Dependence	307	0.000213	5
G1/G1 Phase Transition Leukaemia Cell Lines	ALL	Dependence	310	0.000406	3
Maturation Acute Myeloblastic Leukaemia Cell Lines	AML	Dependence	314	0.000896	2
Interphase Leukaemia Cell Lines		Dependence	319	0.001730	5
Drug Resistance Leukaemia Cell Lines		Dependence	319	0.001770	2
Apoptosis Chronic Myeloid Leukaemia Cell Lines	CML	Dependence	319	0.001770	2
Refractory Chronic Myeloid Leukaemia	CML	Dependence	321	0.001960	4
Non-M3 Acute Myeloid Leukaemia	AML	Dependence	323	0.002570	9
Philadelphia Chromosome Negative Acute Lymphoblastic Leukaemia	ALL	Withdrawal	340	0.000601	4
B-Lymphoblastic Leukaemia / Lymphoma w Recurrent Genetic Abnormalities	ALL	Withdrawal	340	0.006110	5
Acute Leukemia	ALL	Withdrawal	341	0.000626	36
Acute Leukemia	AML	Withdrawal	341	0.000626	36
Precursor B-cell Acute Lymphoblastic Leukaemia	ALL	Withdrawal	341	0.000684	16
Proliferation Leukaemia Cell Lines		Withdrawal	346	0.001870	8
Imatinib Resistant CML in Blast Crisis	CML	Withdrawal	347	0.002120	2
Myelodysplastic Syndrome or Leukaemia		Withdrawal	347	0.002160	40
Acute Lymphoblastic Leukaemia	ALL	Withdrawal	348	0.002480	17
T-Lymphoblastic Leukaemia / Lymphoma	ALL	Withdrawal	348	0.002570	9

Imatinib Resistant CML	CML	Withdrawal	349	0.003080	2
Imatinib Resistant CML in Accelerated Phase	CML	Withdrawal	349	0.003080	2
B-Cell Leukaemia	ALL	Withdrawal	349	0.003110	17
Chemotherapy Refractory ALL	ALL	Withdrawal	350	0.003620	2
Leukaemia		Withdrawal	351	0.003760	38
E2A-PBX B-Cell ALL	ALL	Withdrawal	351	0.004120	3
Philadelphia-Like B-cell ALL	ALL	Withdrawal	352	0.004380	3
KMT2A-Rearrangement B-Cell ALL	ALL	Withdrawal	353	0.004640	3
Accelerated Phase CML	CML	Withdrawal	354	0.005210	3
Philadelphia Positive B-Cell ALL	ALL	Withdrawal	354	0.005820	3
Newly Diagnosed Leukaemia		Withdrawal	356	0.006650	4
Imatinib Resistant Philadelphia positive CML	CML	Withdrawal	356	0.006950	2
B-Cell Non-Hodgkin Lymphoma	NHL	Dependence	303	9.67E-05	45
Non-Hodgkin Lymphoma	NHL	Dependence	307	0.000208	55
Lymphoma - Hodgkins / DLBCNHL	NHL	Dependence	314	0.000777	3
B-Cell Non-Hodgkin Lymphoma	NHL	Withdrawal	354	0.005700	19
T-Lymphoblastic Leukaemia / Lymphoma	NHL	Dependence	300	4.08E-05	20
B-cell Lymphoma	NHL	Dependence	301	5.17E-05	48
Lymphoma	NHL	Dependence	303	0.000101	64
Diffuse Lymphoma	NHL	Dependence	313	0.000629	35
Diffuse B-cell Lymphoma	NHL	Dependence	315	0.000927	32
High Grade Lymphoma	NHL	Dependence	324	0.002690	20
B-Cell Non-Hodgkin Lymphoma Recurrent Genetic Abnormalities	NHL	Withdrawal	340	0.000611	5
B-Cell Lymphoblastic Lymphoma	NHL	Withdrawal	343	0.000891	4
T-Cell Lymphoblastic Leukaemia / Lymphoma	NHL	Withdrawal	348	0.002570	9
KMT2A-Rearrangement B-Cell Lymphoma	NHL	Withdrawal	353	0.004640	3
B-Cell Acute Lymphoblastic Lymphoma	NHL	Withdrawal	355	0.006460	3
Breast / Colorectal cancer	Breast	Dependence	280	3.25E-08	233

Colorectal / Breast cancer	Colorectal	Dependence	280	3.25E-08	233
Breast / Gynaecological cancer	Gynaecological	Dependence	281	1.08E-08	233
Gynaecological / Breast cancer	Gynaecological	Dependence	281	1.08E-08	233
Breast / Pancreatic cancer	Breast	Dependence	290	2.64E-06	181
Pancreatic / Breast cancer	Pancreas	Dependence	290	2.64E-06	181
Breast / Ovarian cancer	Breast	Dependence	304	0.000116	121
Ovarian / Breast	Ovary	Dependence	304	0.000116	121
Breast cancer cell lines morphogenesis	Breast	Dependence	312	0.000573	3
Breast / Ovarian cancer	Breast	Dependence	315	0.000965	133
Ovarian / Breast	Ovary	Dependence	315	0.000965	133
Breast cancer cell lines metastatic potential	Breast	Dependence	316	0.00131	3
Breast cancer cell lines cell proliferation	Breast	Withdrawal	344	0.00127	14
Breast / Gynaecological cancer	Breast	Withdrawal	346	0.00201	91
Gynaecological / Breast cancer	Gynaecological	Withdrawal	346	0.00201	91
Breast / Pancreatic cancer	Breast	Withdrawal	355	0.00592	71
Pancreatic / Breast cancer	Pancreas	Withdrawal	355	0.00592	71
Breast cancer cell lines cell division	Breast	Withdrawal	358	0.00701	1
Genitourinary adenocarcinoma	Urinary	Dependence	259	2.54E-14	259
Genitourinary adenocarcinoma	Urinary	Dependence	271	1.08E-11	277
Malignant genitourinary solid tumour	Urinary	Dependence	277	1.08E-10	277
Genitourinary adenocarcinoma	Urinary	Dependence	277	1.15E-10	277
Urinary tract cancer	Urinary	Dependence	320	0.001890	84
Morphology of urinary system	Urinary	Dependence	322	0.002200	17
Genitourinary adenocarcinoma	Urinary	Withdrawal	336	0.000216	97
Genitourinary adenocarcinoma	Urinary	Withdrawal	338	0.000322	359
Malignant genitourinary solid tumour	Urinary	Withdrawal	339	0.000466	111
Genitourinary tumour	Urinary	Withdrawal	340	0.000547	112
Prostate cancer	Prostate	Dependence	279	2.88E-08	148
Prostatic adenocarcinoma	Prostate	Dependence	287	8.39E-07	123
Prostatic carcinoma	Prostate	Dependence	288	9.39E-07	128

Protate cancer	Prostate	Withdrawal	340	5.33E-04	59
Prostatic adenocarcinoma	Prostate	Withdrawal	350	0.00349	48
Prostatic carcinoma	Prostate	Withdrawal	50	0.00363	50
Prostate cancer cell adhesion	Prostate	Withdrawal	357	0.00701	1
Male Genital Neoplasm	Testis	Dependence	279	1.14E-08	153
Malignant Male Genital Neoplasm	Testis	Dependence	279	1.60E-08	151
Male Genital Neoplasm	Testis	Withdrawal	341	6.75E-04	60
Ovarian cancer	Ovary	Dependence	305	0.000140	68
Ovarian Tumour	Ovary	Dependence	308	0.000257	74
Ovarian cancer	Ovary	Dependence	312	0.000562	71
Ovarian adenocarcinoma	Ovary	Dependence	314	0.000792	62
Ovarian cancer	Ovary	Withdrawal	356	0.007010	1
Brain glioma	Brain	Dependence	284	1.39E-07	227
Brain tumour	Brain	Dependence	286	2.29E-07	228
High grade Astrocytoma	Brain	Dependence	294	5.95E-06	179
Brain astrocytoma	Brain	Dependence	294	6.45E-06	173
Brain cancer	Brain	Dependence	308	0.000269	98
Brain cell - cell movement	Brain	Dependence	319	0.001740	8
Brain astrocytoma	Brain	Withdrawal	330	5.12E-05	77
Brain tumour	Brain	Withdrawal	337	2.56E-04	94
Brain glioma	Brain	Withdrawal	339	0.000520	92
Brain cell - cell movement	Brain	Withdrawal	347	0.002040	5
Glioma	Brain	Dependence	281	5.33E-08	235
Glioma cancer	Brain	Dependence	282	6.69E-08	231
Brain glioma	Brain	Dependence	284	1.39E-07	227
Grade 3-4 Glioma cancer	Brain	Dependence	289	2.18E-06	194
Grade IV High grade glioma cancer	Brain	Dependence	293	5.70E-05	173
Gliomatosis cerebri	Brain	Dependence	307	0.000197	94
Grade IV High grade glioma cancer	Brain	Withdrawal	329	0.000047	77

Glioma	Brain	Withdrawal	330	0.000063	98
Grade 3-4 Glioma cancer	Brain	Withdrawal	337	0.000266	82
Glioma cancer	Brain	Withdrawal	338	0.000305	94
Glioma cancer	Brain	Withdrawal	339	0.000520	92
Astrocytoma	Brain	Dependence	291	3.52E-06	181
Astrocytoma	Brain	Dependence	293	5.70E-06	173
Astrocytoma High grade	Brain	Dependence	294	5.95E-06	179
Astrocytoma	Brain	Dependence	294	6.45E-06	179
Astrocytoma High grade	Brain	Withdrawal	326	8.42E-06	82
Astrocytoma Grade IV	Brain	Withdrawal	329	4.77E-05	77
Astrocytoma	Brain	Withdrawal	330	5.12E-05	77
Colon carcinoma	Colorectal	Dependence	264	7.27E-13	195
Colorectal carcinoma	Colorectal	Dependence	266	1.59E-12	201
Colon carcinoma	Colorectal	Dependence	267	2.04E-12	199
Colon carcinoma	Colorectal	Dependence	268	2.30E-12	197
Colon carcinoma	Colorectal	Dependence	268	2.84E-12	191
Colon carcinoma	Colorectal	Dependence	275	7.38E-11	213
Colon carcinoma	Colorectal	Dependence	278	1.95E-10	214
Colon carcinoma	Colorectal	Withdrawal	341	0.000617	74
Colon carcinoma	Colorectal	Withdrawal	341	0.000687	71
Colon carcinoma	Colorectal	Withdrawal	342	0.000748	70
Colon carcinoma	Colorectal	Withdrawal	342	0.000867	72
Colon carcinoma	Colorectal	Withdrawal	344	0.001290	72
Colon carcinoma	Colorectal	Withdrawal	352	0.004760	78
Rectum tumour	Colorectal	Dependence	311	0.000450	32
Rectum tumour	Colorectal	Dependence	318	0.001550	30
Rectum tumour	Colorectal	Dependence	320	0.001820	29
Rectum tumour	Colorectal	Withdrawal	348	0.002290	15
Renal clear cell carcinoma	Kidney	Dependence	315	0.00101	41
Upper renal tract carcinoma	Kidney	Dependence	324	0.00283	48

Liver carcinoma	Liver	Dependence	283	1.17E-08	168
Liver carcinoma	Liver	Dependence	287	2.52E-07	171
Liver carcinoma	Liver	Dependence	186	5.13E-06	186
Liver tumour	Liver	Dependence	292	5.13E-06	186
Hepatobiliary system cancer	Liver	Dependence	286	2.47E-07	179
Pancreatobiliary tumour	Pancreas	Dependence	288	9.10E-07	124
Pancreatic cancer	Pancreas	Dependence	297	1.19E-05	95
Pancreatic cancer	Pancreas	Dependence	298	1.26E-06	106
Pancreatic cancer	Pancreas	Dependence	299	0.000021	90
Pancreatic cancer	Pancreas	Dependence	300	0.000033	85
Pancreatic cancer	Pancreas	Dependence	301	0.000060	82
Pancreatic cancer cell lines cell migration	Pancreas	Dependence	323	0.002390	6
Pancreatic cancer cell lines cell invasion	Pancreas	Withdrawal	353	0.005210	3
Pancreatic cancer cell lines G1 phase	Pancreas	Withdrawal	355	0.006200	2
Pancreatic cancer	Pancreas	Withdrawal	356	0.00646	36
Melanoma - cutaneous	Melanoma	Dependence	256	3.70E-15	281
Melanoma	Melanoma	Dependence	258	2.36E-14	298
Melanoma	Melanoma	Withdrawal	326	7.71E-06	110
Melanoma	Melanoma	Withdrawal	330	6.21E-05	115
Myeloma - cell lines cell death	Myeloma	Withdrawal	345	0.00159	5
Myeloma - cell lines apoptosis	Myeloma	Withdrawal	355	0.00622	4
Myeloma - cell lines cell adhesion	Myeloma	Withdrawal	357	0.00701	1
Gastroesophageal carcinoma	Stomach	Dependence	262	3.15E-13	169
Gastroesophageal carcinoma	Esophagus	Dependence	262	3.15E-13	169
Gastroesophageal carcinoma	Stomach	Dependence	265	1.53E-12	171
Gastroesophageal carcinoma	Esophagus	Dependence	265	1.53E-12	171
Gastroesophageal carcinoma	Stomach	Dependence	282	9.77E-08	103

Gastroesophageal carcinoma	Esophagus	Dependence	282	9.77E-08	103
Esophageal adenocarcinoma	Esophagus	Dependence	320	0.001880	22
Gastroesophageal carcinoma	Stomach	Withdrawal	331	0.000068	64
Gastroesophageal carcinoma	Esophagus	Withdrawal	331	0.000068	64
Esophageal carcinoma	Esophagus	Withdrawal	347	0.002020	15
Gastroesophageal adenocarcinoma	Esophagus	Withdrawal	354	0.005250	38
Gastroesophageal adenocarcinoma	Stomach	Withdrawal	354	0.005250	38
Upper aerodigestive tract carcinoma	Oropharynx	Dependence	283	1.25E-07	97
Upper aerodigestive tract carcinoma	Oropharynx	Dependence	285	1.82E-06	98
Upper aerodigestive tract carcinoma	Oropharynx	Withdrawal	327	1.60E-05	44
