# Supplementary material for: Novel Insights into Potential Cannabis-Related Cancerogenesis from Recent Key Whole Epigenome Screen of Cannabis Dependence and Withdrawal: Epidemiological Commentary and Explication of Schrott et al
Source: Genes (Basel). 2022 Dec 22;14(1):32. doi: 10.3390/genes14010032 (PMC9858221; doi:10.3390/genes14010032)
Supplement: Supplementary file 1 [file genes-14-00032-s001.zip › R_Code for Cancer Epigenomics Paper.pdf]

```
library(tidyverse)
library(broom)
library(broom.mixed)
library(viridis)
library(ggpubr)
library(albersusa)
library(EValue)
library(epiR)
library(tibble)
library(sf)
library(nlme)
library(plm)
library(ggpubr)
library(margins)
library(ggrepel)
library(scales)
library(tidyverse)



setwd("D:/1ASReece/Research/2020 Res/Book - CI-HCE/Rev 1/R Codes")
getwd()
## Use Control+L to empty Console if too slow....


########## Read-In's
CaEpiD <- read_csv("D:/1ASReece/Research/2022 Res/Cancer Epigenomics/R
Codes/Data/csv/CaEpi.csv")

CaEpiDMdn       <- read_csv("D:/1ASReece/Research/2022 Res/Cancer Epigenomics/R
Codes/Data/csv/CaEpiDMdn.csv")
CaEpiDMdnMn     <- read_csv("D:/1ASReece/Research/2022 Res/Cancer Epigenomics/R
Codes/Data/csv/CaEpiDMdnMn.csv")
CaEpiDMdnStat   <- read_csv("D:/1ASReece/Research/2022 Res/Cancer Epigenomics/R
Codes/Data/csv/CaEpiDMdnStat.csv")

CaEpiDMin       <- read_csv("D:/1ASReece/Research/2022 Res/Cannabis_Ageing/R
Codes/Data/csv/CaEpiDMin.csv")
CaEpiDMinMn     <- read_csv("D:/1ASReece/Research/2022 Res/Cannabis_Ageing/R
Codes/Data/csv/CaEpiDMinMn.csv")
CaEpiDMinStat   <- read_csv("D:/1ASReece/Research/2022 Res/Cannabis_Ageing/R
Codes/Data/csv/CaEpiDMinStat.csv")


dim(CaEpiD); names(CaEpiD); head(CaEpiD)
class(CaEpiD)
summary(CaEpiD); glimpse(CaEpiD)

unique(CaEpiD$Canc)
length(unique(CaEpiD$Canc))
```

```
table(CaEpiD$Canc)
table(CaEpiD$Status)

hist(CaEpiD$P_Value)
hist(log(CaEpiD$P_Value))

shapiro.test(CaEpiD$P_Value)       #         ## W = 0.70875, p-value < 2.2e-16
shapiro.test(log(CaEpiD$P_Value))  # log it  ## W = 0.76197, p-value = 3.567e-15

shapiro.test(CaEpiD$Gene_Number)       #         ## W = 0.89907, p-value = 2.836e-09
shapiro.test(log(CaEpiD$Gene_Number))  # Don't   ## W = 0.87947, p-value = 2.32e-10

hist(CaEpiD$Gene_Number)
hist(log(CaEpiD$Gene_Number))


### Medians
CaEpiDMdn <- CaEpiD %>%
  mutate(Row_P  = P_Value * Gene_Number) %>%
  group_by(Canc) %>%
  summarize(
    Number_Annotations = n(),
    mdnP_Value     = median(P_Value),
    mdnGene_Number = median(Gene_Number),
    CumvGeneNo     = sum(Gene_Number),
    Cumv_P         = sum(Row_P),
    mnP_Value      = Cumv_P / CumvGeneNo
  ) %>% arrange(mdnP_Value)
CaEpiDMdn

CaEpiDMdnMn <- CaEpiDMdn %>% arrange(mnP_Value)
CaEpiDMdnMn

### Medians by Dependence / Withdrawal Status
CaEpiDMdnStat <- CaEpiD %>%
  group_by(Status, Canc) %>%
  summarize(
    mdnP_Value     = median(P_Value),
    mdnGene_Number = median(Gene_Number)
  ) %>%
  pivot_wider(
    names_from = Status,
    values_from = c(mdnP_Value, mdnGene_Number)
  ) %>%
  mutate(
    PVal_Ratio = mdnP_Value_Withdrawal / mdnP_Value_Dependence,
    GenNoRatio = mdnGene_Number_Dependence / mdnGene_Number_Withdrawal
  ) %>%
  arrange(mdnP_Value_Dependence) %>%
  .[,c(1:3,6,4,5,7)]
```

```
CaEpiDMdnStat


write_csv(CaEpiDMdn, "D:/1ASReece/Research/2022 Res/Cancer Epigenomics/R
Codes/Data/csv/CaEpiDMdn.csv")
write_csv(CaEpiDMdnMn, "D:/1ASReece/Research/2022 Res/Cancer Epigenomics/R
Codes/Data/csv/CaEpiDMdnMn.csv")
write_csv(CaEpiDMdnStat, "D:/1ASReece/Research/2022 Res/Cancer Epigenomics/R
Codes/Data/csv/CaEpiDMdnStat.csv")


### Minimums
CaEpiDMin <- CaEpiD %>%
  mutate(Row_P  = P_Value * Gene_Number) %>%
  group_by(Canc) %>%
  summarize(
    Number_Annotations = n(),
    minP_Value     = min(P_Value),
    # mdnGene_Number = median(Gene_Number),
    CumvGeneNo     = sum(Gene_Number),
    Cumv_P         = sum(Row_P),
    mnP_Value      = Cumv_P / CumvGeneNo
  ) %>% arrange(minP_Value)
CaEpiDMin

CaEpiDMinMn <- CaEpiDMin %>% arrange(mnP_Value)
CaEpiDMinMn

### Minimums by Dependence / Withdrawal Status
CaEpiDMinStat <- CaEpiD %>%
  group_by(Status, Canc) %>%
  summarize(
    minP_Value     = min(P_Value),
    totGene_Number = sum(Gene_Number)
  ) %>%
  pivot_wider(
    names_from = Status,
    values_from = c(minP_Value, totGene_Number)
  ) %>%
  mutate(
    PVal_Ratio = minP_Value_Withdrawal / minP_Value_Dependence,
    GenNoRatio = totGene_Number_Dependence / totGene_Number_Withdrawal
  ) %>%
  arrange(minP_Value_Dependence) %>%
  .[,c(1:3,6,4,5,7)]
CaEpiDMinStat


write_csv(CaEpiDMin, "D:/1ASReece/Research/2022 Res/Cannabis_Ageing/R
Codes/Data/csv/CaEpiDMin.csv")
```

```
write_csv(CaEpiDMinMn, "D:/1ASReece/Research/2022 Res/Cannabis_Ageing/R Codes/Data/csv/CaEpiDMinMn.csv")
write_csv(CaEpiDMinStat, "D:/1ASReece/Research/2022 Res/Cannabis_Ageing/R Codes/Data/csv/CaEpiDMinStat.csv")


########################################################################################
########################################################################################
############## Graphs


# mdnP_Value - Overall
mdnP_Value <-
ggplot(CaEpiDMdn, aes(reorder(Canc, desc(mdnP_Value)), -log10(mdnP_Value), group = reorder(Canc, desc(mdnP_Value)),
                 fill = reorder(Canc, desc(mdnP_Value)), colour = reorder(Canc, desc(mdnP_Value)),
                 label = round(-log10(mdnP_Value),2) )) +
  geom_col(alpha = 0.75, size = 1.5, show.legend = F) +
  ggtitle ('Negative Log Median P-Value by Cancer Type') +
  labs(x='', y = '- Log (Median P-Value)') +
  theme_plotF() +
  geom_text(nudge_y = 1, show.legend = F) +
  theme(axis.text.x=element_text(angle=30,hjust=1),
      plot.margin = unit(c(0.5,0.5,0.5,2), "cm"))

ggsave("Cancer Epigenomic Median P-Values_2.pdf", width=12, height = 6, dpi=1200, units="in",
     path= "D:/1ASReece/Research/2022 Res/Cancer Epigenomics/R Codes/Data/Figures")


# mdnGene_Number - Overall
mdnGeneNo <-
ggplot(CaEpiDMdn, aes(reorder(Canc, (mdnGene_Number)), (mdnGene_Number), group = reorder(Canc, (mdnGene_Number)),
                 fill = reorder(Canc, (mdnGene_Number)), colour = reorder(Canc, (mdnGene_Number)),
                 label = mdnGene_Number)) +
  geom_col(alpha = 0.75, size = 1.5, show.legend = F) +
  geom_text(nudge_y = 10, show.legend = F) +
  ggtitle ('Median Gene Number by Cancer Type') +
  labs(x='', y = 'Gene Number') +
  theme_plotF() +
  theme(axis.text.x=element_text(angle=30,hjust=1),
      plot.margin = unit(c(0.5,0.5,0.5,2), "cm"))

ggsave("Cancer Epigenomic Median Gene Number.pdf", width=12, height = 6, dpi=1200, units="in",
```

```
     path= "D:/1ASReece/Research/2022 Res/Cancer Epigenomics/R Codes/Data/Figures")


PGeneNo <- ggarrange(mdnGeneNo, mdnP_Value, nrow=2, labels = "AUTO")
PGeneNo

ggsave("Cancer Epigenomic Median & Gene Number.pdf", width=12, height = 10,
dpi=1200, units="in",
     path= "D:/1ASReece/Research/2022 Res/Cancer Epigenomics/R Codes/Data/Figures")

ggsave("Fig. 1 - Cancer Epigenomic Median & Gene Number - Overall.pdf.pdf", width=9,
height = 12, dpi=1200, units="in",
     path= "D:/1ASReece/Research/2022 Res/Cancer Epigenomics/Journal/Intl J
Cancer/Figg._New")


### Now Looking at Dependence v Withdrawal

# mdnP_Value - Dependence
DepP <-
ggplot(CaEpiDMdnStat[-20,], aes(reorder(Canc, desc(mdnP_Value_Dependence)), -
log10(mdnP_Value_Dependence),
                   group = reorder(Canc, desc(mdnP_Value_Dependence)),
                fill = reorder(Canc, desc(mdnP_Value_Dependence)),
                colour = reorder(Canc, desc(mdnP_Value_Dependence)),
                label = round(-log10(mdnP_Value_Dependence),2) )) +
  geom_col(alpha = 0.75, size = 1.5, show.legend = F) +
  ggtitle ('Negative Log Median P-Value by Cancer Type in Cannabis Dependence') +
  labs(x='', y = '- Log (Median P-Value)') +
  theme_plotF() +
  geom_text(nudge_y = 1, show.legend = F) +
  theme(axis.text.x=element_text(angle=30,hjust=1),
      plot.margin = unit(c(0.5,0.5,0.5,2), "cm"))

ggsave("Cancer Epigenomic Median P-Values Dependence_2.pdf", width=12, height = 6,
dpi=1200, units="in",
     path= "D:/1ASReece/Research/2022 Res/Cancer Epigenomics/R Codes/Data/Figures")

# mdnGene_Number - Dependence
GeneNoDep <-
ggplot(CaEpiDMdnStat[-20,], aes(reorder(Canc, (mdnGene_Number_Dependence)),
(mdnGene_Number_Dependence),
                group = reorder(Canc, (mdnGene_Number_Dependence)),
                fill = reorder(Canc, (mdnGene_Number_Dependence)),
                colour = reorder(Canc, (mdnGene_Number_Dependence)),
                label = mdnGene_Number_Dependence)) +
  geom_col(alpha = 0.75, size = 1.5, show.legend = F) +
  geom_text(nudge_y = 10, show.legend = F) +
  ggtitle ('Median Gene Number by Cancer Type in Cannabis Dependence') +
  labs(x='', y = 'Gene Number') +
```

```
  theme_plotF() +
  theme(axis.text.x=element_text(angle=30,hjust=1),
        plot.margin = unit(c(0.5,0.5,0.5,2), "cm"))

ggsave("Cancer Epigenomic Median Gene Number Dependence.pdf", width=12, height = 6, dpi=1200, units="in",
       path= "D:/1ASReece/Research/2022 Res/Cancer Epigenomics/R Codes/Data/Figures")


PGeneNoDep <- ggarrange(GeneNoDep, DepP, nrow=2, labels = "AUTO")
PGeneNoDep

ggsave("Cancer Epigenomic Median & Gene Number - Dependence.pdf", width=12, height = 10, dpi=1200, units="in",
       path= "D:/1ASReece/Research/2022 Res/Cancer Epigenomics/R Codes/Data/Figures")

ggsave("Fig. 2 - Cancer Epigenomic Median & Gene Number - Dependence.pdf.pdf", width=9, height = 12, dpi=1200, units="in",
       path= "D:/1ASReece/Research/2022 Res/Cancer Epigenomics/Journal/Intl J Cancer/Figg._New")


##### Now Ratios Dependence to Withdrawal

# mdnP_Value - Dependence
PRatioDep <-
ggplot(CaEpiDMdnStat[-c(8,19,20),], aes(reorder(Canc, (PVal_Ratio)), log(PVal_Ratio),
                              group = reorder(Canc, (PVal_Ratio)),
                              fill = reorder(Canc, (PVal_Ratio)),
                              colour = reorder(Canc, (PVal_Ratio)),
                              label = round(log(PVal_Ratio),2) )) +
  geom_col(alpha = 0.75, size = 1.5, show.legend = F) +
  ggtitle ('Median P-Value Dependence / Withdrawal Ratio by Cancer Type') +
  labs(x='', y = 'Log (Ratio of Median P-Values Dependence to Withdrawal)') +
  theme_plotF() +
  geom_text(nudge_y = 1.5, show.legend = F) +
  theme(axis.text.x=element_text(angle=30,hjust=1),
        plot.margin = unit(c(0.5,0.5,0.5,2), "cm"))

ggsave("Cancer Epigenomic Median P-Values Dep-With Ratio.pdf", width=12, height = 6, dpi=1200, units="in",
       path= "D:/1ASReece/Research/2022 Res/Cancer Epigenomics/R Codes/Data/Figures")

# mdnGene_Number - Dependence
GeneRatioDep <-
ggplot(CaEpiDMdnStat[-c(8,19,20),], aes(reorder(Canc, (GenNoRatio)), log(GenNoRatio),
                              group = reorder(Canc, (GenNoRatio)),
                              fill = reorder(Canc, (GenNoRatio)),
                              colour = reorder(Canc, (GenNoRatio)),
                              label = round(GenNoRatio,2)  )) +
```

```
  geom_col(alpha = 0.75, size = 1.5, show.legend = F) +
  geom_text(nudge_y = 0.3, show.legend = F) +
  ggtitle ('Log (Ratio (Median Gene Number (Dependence / Withdrawal) Ratio by Cancer
Type') +
  labs(x='', y = 'Log (Gene Number Ratio (Dependence / Withdrawal)') +
  theme_plotF() +
  theme(axis.text.x=element_text(angle=30,hjust=1),
        plot.margin = unit(c(0.5,0.5,0.5,2), "cm"))

ggsave("Cancer Epigenomic Median Gene Number Dep-With Ratio.pdf", width=12, height =
6, dpi=1200, units="in",
       path= "D:/1ASReece/Research/2022 Res/Cancer Epigenomics/R Codes/Data/Figures")

PGeneNoDepRatio <- ggarrange(GeneRatioDep, PRatioDep, nrow=2, labels = "AUTO")
PGeneNoDepRatio

ggsave("Cancer Epigenomic Median & Gene Number - Ratios.pdf", width=12, height = 10,
dpi=1200, units="in",
       path= "D:/1ASReece/Research/2022 Res/Cancer Epigenomics/R Codes/Data/Figures")

ggsave("Fig. 3 - Cancer Epigenomic Median & Gene Number - Ratios.pdf", width=9, height
= 12, dpi=1200, units="in",
       path= "D:/1ASReece/Research/2022 Res/Cancer Epigenomics/Journal/Intl J
Cancer/Figg._New")



################################################################################
####
################################################################################
####
################################################################################
####
####### New Graphs

# minP_Value - Overall
minP <-
ggplot(CaEpiDMin, aes(reorder(Canc, desc(minP_Value)), -log(minP_Value), group =
reorder(Canc, desc(minP_Value)),
                fill = reorder(Canc, desc(minP_Value)), colour = reorder(Canc,
desc(minP_Value)),
                label = round(-log(minP_Value),2) )) +
  geom_col(alpha = 0.75, size = 1.5, show.legend = F) +
  ggtitle ('Negative Log Minimum P-Value by Cancer Type') +
  labs(x='', y = '- Log (Minimum P-Value)') +
  theme_plotF() +
  geom_text(nudge_y = 2, show.legend = F) +
  theme(axis.text.x=element_text(angle=30,hjust=1),
        plot.margin = unit(c(0.5,0.5,0.5,2), "cm"))
```

```
ggsave("Cancer Epigenomic Minimum P-Values_2.pdf", width=12, height = 6, dpi=1200,
units="in",
      path= "D:/1ASReece/Research/2022 Res/Cannabis_Ageing/R Codes/Data/Figures")


# No. Annotations
NoAnnot <-
  ggplot(CaEpiDMin, aes(reorder(Canc, (Number_Annotations )), (Number_Annotations ),
                     group = reorder(Canc, (Number_Annotations )),
                     fill = reorder(Canc, (Number_Annotations )),
                     colour = reorder(Canc, (Number_Annotations )),
                     label = round((Number_Annotations ),2) )) +
  geom_col(alpha = 0.75, size = 1.5, show.legend = F) +
  ggtitle ('Number of Annotations by Cancer Type') +
  labs(x='', y = 'Number of Annotations') +
  theme_plotF() +
  geom_text(nudge_y = 1, show.legend = F) +
  theme(axis.text.x=element_text(angle=30,hjust=1),
       plot.margin = unit(c(0.5,0.5,0.5,2), "cm"))

ggsave("Number Annotations.pdf", width=12, height = 6, dpi=1200, units="in",
      path= "D:/1ASReece/Research/2022 Res/Cannabis_Ageing/R Codes/Data/Figures")


# Cumulative Gene Number
CumGeneNo <-
  ggplot(CaEpiDMin, aes(reorder(Canc, (CumvGeneNo  )), (CumvGeneNo),
                     group = reorder(Canc, (CumvGeneNo  )),
                     fill = reorder(Canc, (CumvGeneNo  )),
                     colour = reorder(Canc, (CumvGeneNo  )),
                     label = round((CumvGeneNo  ),2) )) +
  geom_col(alpha = 0.75, size = 1.5, show.legend = F) +
  ggtitle ('Cumulative Gene Number by Cancer Type') +
  labs(x='', y = 'Cumulative Gene Number') +
  theme_plotF() +
  geom_text(nudge_y = 250, show.legend = F) +
  theme(axis.text.x=element_text(angle=30,hjust=1),
       plot.margin = unit(c(0.5,0.5,0.5,2), "cm"))

ggsave("Number Annotations.pdf", width=12, height = 6, dpi=1200, units="in",
      path= "D:/1ASReece/Research/2022 Res/Cannabis_Ageing/R Codes/Data/Figures")


minAnnotGene <- ggarrange(CumGeneNo, NoAnnot, nrow=2, labels = "AUTO")
minAnnotGene

ggsave("Gene & Annoation Numbers.pdf", width=12, height = 10, dpi=1200, units="in",
      path= "D:/1ASReece/Research/2022 Res/Cannabis_Ageing/R Codes/Data/Figures")
```

```
minAnnotGeneP <- ggarrange(NoAnnot , CumGeneNo, minP, nrow=3, labels = "AUTO")
minAnnotGeneP

ggsave("Gene & Annoation Numbers & P's_2.pdf", width=12, height = 14, dpi=1200,
units="in",
      path= "D:/1ASReece/Research/2022 Res/Cannabis_Ageing/R Codes/Data/Figures")


### Now Looking at Dependence v Withdrawal

# minP_Value - Dependence
DepP <-
  ggplot(CaEpiDMinStat[-20,], aes(reorder(Canc, desc(minP_Value_Dependence)), -
log(minP_Value_Dependence),
                              group = reorder(Canc, desc(minP_Value_Dependence)),
                              fill = reorder(Canc, desc(minP_Value_Dependence)),
                              colour = reorder(Canc, desc(minP_Value_Dependence)),
                              label = round(-log(minP_Value_Dependence),2) )) +
  geom_col(alpha = 0.75, size = 1.5, show.legend = F) +
  ggtitle ('Negative Log of Minimum P-Value by Cancer Type in Cannabis Dependence') +
  labs(x='', y = '- Log (Minimum P-Value)') +
  theme_plotF() +
  geom_text(nudge_y = 1.2, show.legend = F) +
  theme(axis.text.x=element_text(angle=30,hjust=1),
       plot.margin = unit(c(0.5,0.5,0.5,2), "cm"))

ggsave("Cancer Epigenomic Minimum P-Values Dependence.pdf", width=12, height = 6,
dpi=1200, units="in",
      path= "D:/1ASReece/Research/2022 Res/Cannabis_Ageing/R Codes/Data/Figures")

# totGene_Number - Dependence
GeneNoDep <-
  ggplot(CaEpiDMinStat[-20,], aes(reorder(Canc, (totGene_Number_Dependence)),
(totGene_Number_Dependence),
                              group = reorder(Canc, (totGene_Number_Dependence)),
                              fill = reorder(Canc, (totGene_Number_Dependence)),
                              colour = reorder(Canc, (totGene_Number_Dependence)),
                              label = totGene_Number_Dependence)) +
  geom_col(alpha = 0.75, size = 1.5, show.legend = F) +
  geom_text(nudge_y = 200, show.legend = F) +
  ggtitle ('Total Gene Number by Cancer Type in Cannabis Dependence') +
  labs(x='', y = 'Gene Number') +
  theme_plotF() +
  theme(axis.text.x=element_text(angle=30,hjust=1),
       plot.margin = unit(c(0.5,0.5,0.5,2), "cm"))

ggsave("Total Epigenomic Minimum Gene Number Dependence.pdf", width=12, height = 6,
dpi=1200, units="in",
      path= "D:/1ASReece/Research/2022 Res/Cancer Epigenomics/R Codes/Data/Figures")
```

```
PGeneNoDep <- ggarrange(GeneNoDep, DepP, nrow=2, labels = "AUTO")
PGeneNoDep

ggsave("Cancer Epigenomic Median & Gene Number - Dependence_2.pdf", width=12,
height = 10, dpi=1200, units="in",
      path= "D:/1ASReece/Research/2022 Res/Cannabis_Ageing/R Codes/Data/Figures")



##### Now Ratios Dependence to Withdrawal

# minP_Value - Dependence
PRatioDep <-
  ggplot(CaEpiDMinStat[-c(8,19,20),], aes(reorder(Canc, (PVal_Ratio)), log10(PVal_Ratio),
                                group = reorder(Canc, (PVal_Ratio)),
                                fill = reorder(Canc, (PVal_Ratio)),
                                colour = reorder(Canc, (PVal_Ratio)),
                                label = round(log10(PVal_Ratio),2) )) +
  geom_col(alpha = 0.75, size = 1.5, show.legend = F) +
  ggtitle ('Log (Ratio (Minimum P-Value (Dependence / Withdrawal))) by Cancer Type') +
  labs(x='', y = 'Log (Ratio of Minimum P-Values Dependence to Withdrawal)') +
  theme_plotF() +
  geom_text(nudge_y = 0.65, show.legend = F) +
  theme(axis.text.x=element_text(angle=30,hjust=1),
       plot.margin = unit(c(0.5,0.5,0.5,2), "cm"))

ggsave("Cancer Epigenomic Minimum P-Values Dep-With Ratio_2.pdf", width=12, height =
6, dpi=1200, units="in",
      path= "D:/1ASReece/Research/2022 Res/Cannabis_Ageing/R Codes/Data/Figures")

# minGene_Number - Dependence
GeneRatioDep <-
  ggplot(CaEpiDMinStat[-c(8,19,20),], aes(reorder(Canc, (GenNoRatio)),
log10(GenNoRatio),
                                group = reorder(Canc, (GenNoRatio)),
                                fill = reorder(Canc, (GenNoRatio)),
                                colour = reorder(Canc, (GenNoRatio)),
                                label = round(GenNoRatio,2)  )) +
  geom_col(alpha = 0.75, size = 1.5, show.legend = F) +
  geom_text(nudge_y = 0.2, show.legend = F) +
  ggtitle ('Log (Total Gene Number (Dependence / Withdrawal) Ratio) by Cancer Type') +
  labs(x='', y = 'Log (Total Number Ratio (Dependence / Withdrawal))') +
  theme_plotF() +
  theme(axis.text.x=element_text(angle=30,hjust=1),
       plot.margin = unit(c(0.5,0.5,0.5,2), "cm"))

ggsave("Cancer Epigenomic Total Gene Number Dep-With Ratio.pdf", width=12, height = 6,
dpi=1200, units="in",
```

```
      path= "D:/1ASReece/Research/2022 Res/Cannabis_Ageing/R Codes/Data/Figures")

PGeneNoDepRatio <- ggarrange(GeneRatioDep, PRatioDep, nrow=2, labels = "AUTO")
PGeneNoDepRatio

ggsave("Cancer Epigenomic Median & Gene Number - Ratios.pdf", width=12, height = 10,
dpi=1200, units="in",
      path= "D:/1ASReece/Research/2022 Res/Cannabis_Ageing/R Codes/Data/Figures")



CaEpiDMinStat <- CaEpiDMinStat %>% mutate(InvGeneNumberRatio =
totGene_Number_Withdrawal / totGene_Number_Dependence)


# minGene_Number - Dependence
GeneRatioDepInv  <-
  ggplot(CaEpiDMinStat[-c(8,19,20),], aes(reorder(Canc, (InvGeneNumberRatio)),
log10(InvGeneNumberRatio),
                                    group = reorder(Canc, (InvGeneNumberRatio)),
                                    fill = reorder(Canc, (InvGeneNumberRatio)),
                                    colour = reorder(Canc, (InvGeneNumberRatio)),
                                    label = round(InvGeneNumberRatio,2)  )) +
  geom_col(alpha = 0.75, size = 1.5, show.legend = F) +
  geom_text(nudge_y = 0.15, show.legend = F, colour = 'grey10') +
  ggtitle ('Log (Total Gene Number (Withdrawal / Dependence) Ratio) by Cancer Type') +
  labs(x='', y = 'Log (Total Number Ratio (Withdrawal / Dependence))') +
  theme_plotF() +
  theme(axis.text.x=element_text(angle=30,hjust=1),
       plot.margin = unit(c(0.5,0.5,0.5,2), "cm"))

ggsave("Cancer Epigenomic Total Gene Number With-Dep Ratio.pdf", width=12, height = 6,
dpi=1200, units="in",
      path= "D:/1ASReece/Research/2022 Res/Cannabis_Ageing/R Codes/Data/Figures")

PGeneNoDepRatioWith <- ggarrange(PRatioDep, GeneRatioDep, GeneRatioDepInv,
nrow=3, labels = "AUTO")
PGeneNoDepRatioWith

ggsave("Cancer Epigenomic Total Gene Number With-Dep Dep-With Ratio_3.pdf",
width=12, height = 14, dpi=1200, units="in",
      path= "D:/1ASReece/Research/2022 Res/Cannabis_Ageing/R Codes/Data/Figures")
```
